# Elucidation of Expression Patterns and Functional Properties of Archaerhodopsin Derived from *Halorubrum* sp. Ejinoor

**DOI:** 10.3390/biology14040360

**Published:** 2025-03-31

**Authors:** Luomeng Chao, Yuxia Yang

**Affiliations:** 1 College of Animal Science and Technology, Inner Mongolia MINZU University, Tongliao 028000, China; chaoluomen@126.com; 2 College of Computer Science and Technology, Inner Mongolia MINZU University, Tongliao 028000, China

**Keywords:** *Halorubrum* sp. Ejinoor Archaerhodopsin, light dark adaptation, photochemical reaction cycle, proton pump

## Abstract

This study elucidates the structural determinants and optogenetic potential of Archaerhodopsin *He*AR, a proton pump from *Halorubrum* sp. Ejinoor isolated from Inner Mongolian salt lakes. Through heterologous expression in *E. coli* BL21 (DE3) and integrative biophysical analyses, we demonstrate that *He*AR adopts a stable trimeric architecture (129 kDa) with detergent-binding characteristics mirroring bacteriorhodopsin (BR); however, it exhibits a 10 nm bathochromic spectral shift (λmax = 550 nm) and elevated proton affinity (Asp-95 pKa = 3.5 vs. BR Asp-85 pKa = 2.6), indicative of evolutionary optimization in its retinal-binding electrostatic microenvironment. Kinetic profiling reveals *He*AR’s prolonged photocycle (100 ms vs. BR’s 11 ms), marked by rapid M-state decay (3.3 ms) and extended dark-adaptation half-life (160 min), a bistable behavior attributed to enhanced hydrogen bond persistence (80%) and reduced conformational entropy (RMSD = 2.0 Å). Functional assays confirm light-driven proton extrusion (0.1 ng H⁺/mg·s) with DCCD-amplified flux (0.3 ng H⁺/mg·s) and ATP synthesis (0.3 nmol/mg·s), underscoring its synergy with H⁺-ATPase. Phylogenetic and structural analyses reveal 95% homology with *Halorubrum* AR4 and conservation of 11 proton-wire residues, despite divergent Trp/Tyr/Ser networks that redefine chromophore stabilization. AlphaFold-predicted models (TM-score > 0.92) and molecular docking identify superior retinoid-binding affinity (ΔG = −12.27 kcal/mol), while spectral specificity (550–560 nm) and acid-stable photoresponse highlight its adaptability for low-irradiance neuromodulation. These findings position *He*AR as a precision optogenetic tool, circumventing spectral overlap with excitatory opsins and enabling sustained hyperpolarization with minimized phototoxicity. By bridging microbial energetics and optobioengineering, this work expands the archaeal rhodopsin toolkit and provides a blueprint for designing wavelength-optimized photoregulatory systems.

## Simple Summary

This study introduces Archaerhodopsin (*He*AR), a light-driven proton pump from the salt-tolerant archaeon *Halorubrum* sp. Ejinoor, as a next-generation optogenetic tool. *He*AR shares structural similarities with bacteriorhodopsin (BR), forming stable trimers, but exhibits a red-shifted absorption peak (550 nm) and prolonged dark-adaptation (160 min), enabling sustained neuronal inhibition with minimal phototoxicity. Through AlphaFold modeling and biophysical assays, we demonstrate *He*AR’s optimized retinal-binding architecture, which enhances proton-pumping efficiency and stability under physiological conditions. Unlike existing tools, *He*AR’s activation window (550–560 nm) avoids spectral overlap with excitatory opsins, permitting multi-channel neural control. Discovered in Inner Mongolian salt lakes, *He*AR bridges extremophile adaptations and optogenetic engineering, offering a robust platform for non-invasive neuromodulation and therapeutic development. Its unique properties address key limitations in current optogenetics, such as photodamage and temporal precision, positioning *He*AR as a transformative tool for neuroscience research.

## 1. Introduction

Bacteriorhodopsin (BR) is a light-sensitive membrane protein sourced from the highly salt-loving bacterium *Halobacterium salinarum* (*Hs*) [1]. BR is made up of seven transmembrane α-helices connected by loops on both the inside and outside of the cell. The chromophore retinal is covalently attached to the amino acid K216 on the seventh α-helix, forming a Schiff base, and has a molecular weight of 26 kDa [2,3]. BR acts as a light-driven proton pump, synthesizing ATP from a proton gradient to support the growth and development of the bacterium [4]. The abundant expression of BR in *Hs* gives the bacterium its purple color, which is why BR is often referred to as the purple membrane [1]. BR in the *Hs* membrane binds with phospholipids to form a unique trimeric structure, which can be purified using the sucrose gradient method [5]. The purple membrane has a broad absorption spectrum in the visible region, and upon absorption of a photon, retinal undergoes isomerization, resulting in the formation of several intermediate states, K, L, M, N, and O, before returning to the ground state to complete the photochemical reaction cycle [6]. BR can absorb photons and convert energy, playing a crucial role in the survival and adaptation of the bacterium. Due to its simple structure, functional diversity, and wide range of applications, BR has attracted significant attention. Research indicates that BR has promising applications in biophysical technologies [7,8], including the photocatalytic breakdown of organic pollutants, and it serves as an essential tool in optogenetics for processing light signals [9,10,11].

Optogenetics is a research method in cellular biology that merges optics with genetics [12]. Researchers use genetic engineering to introduce light-sensitive proteins into cells, allowing them to activate or inhibit specific cells with light to better understand their functions. Genes that encode light-sensitive proteins are usually transferred to target cells using methods like transfection, viral transduction, or by creating transgenic animals, which help regulate light signals and observe cellular behaviors and functions [13]. Due to its many advantages, such as being non-invasive, having high spatial and temporal resolution as well as quantitative repeatability, and being simple to use, optogenetics has gained widespread attention in neuroscience, molecular biology, and medicine [14,15,16]. Commonly used light-sensitive proteins in optogenetics research include Channelrhodopsin-2 (ChR2) from the green alga Chlamydomonas Reinhardtii and halorhodopsin (*Np*HR) from the halophilic archaeon *Natronomonas pharaonis* [17,18]. ChR2 has an absorption wavelength range of 350–550 nm with a peak absorption wavelength of 470 nm, while *Np*HR has an absorption wavelength range of 525–620 nm with a peak absorption wavelength of 578 nm, indicating different sensitivities to light of different wavelengths between the two proteins. Light at 470 nm and 578 nm does not penetrate tissues well, making it challenging to directly activate light-sensitive proteins in deeper cells. To address this problem, researchers discovered a light-sensitive protein from the primitive green alga Mesostigma viride, which has a maximum absorption wavelength of 530 nm [19]. Additionally, genetic modifications have been made to VChR1 (Channelrhodopsin from *Volvox carteri*) to produce a light-sensitive protein with an absorption wavelength in the range of 590–630 nm [20]. Apart from ChR2 and *Np*HR, the most commonly used BR class protein, Archaerhodopsin 3, which can be activated by red light, pumps protons from the inside to the outside of cells, hyperpolarizes the cell membrane, and inhibits cell excitability [20]. Due to the unique biological functions of bacterial rhodopsin proteins, they play an important role in the field of optogenetics. However, as important tools in optogenetics, the search for light-sensitive proteins that meet the requirements and are highly efficient is crucial [21,22].

This study focuses on the *Halorubrum* sp. Ejinoor Archaerhodopsin(*He*AR). *He*AR was discovered in halophilic bacteria from a salt lake in Inner Mongolia. It features seven transmembrane α-helical regions and contains highly conserved amino acids associated with proton pumping. *He*AR exhibits unique optogenetic advantages over existing Archaerhodopsins: (1) a red-shifted activation window (550–560 nm) minimizing spectral overlap with excitatory opsins; (2) prolonged dark-adaptation (160 min) enabling sustained neuronal silencing; (3) enhanced retinal-binding stability (ΔG = −12.27 kcal/mol) via Trp/Tyr/Ser triad interactions. These traits, coupled with 95% homology to *Halorubrum* AR4 but distinct from BR (57%), position *He*AR as an optogenetic tool for low-irradiance applications.

In this study, *He*AR was expressed in the *E. coli* BL21 (DE3) expression system prior to the analysis of its biological functions. The biological functions of *He*AR were analyzed using SDS-PAGE, UV-visible absorption spectroscopy, CD spectroscopy, laser flash photolysis, and proton pump activity assays. Following purification, size-exclusion chromatography (SEC) revealed that *He*AR predominantly exists as a trimer in physiological buffer conditions. To elucidate the structural basis of this oligomerization, we predicted the *He*AR tertiary structure using AlphaFold. The predicted model demonstrates remarkable topological conservation with BR, particularly in the retinal-binding pocket and proton translocation channels. To validate the structural compatibility between *He*AR and its cognate chromophore, we performed molecular docking, and molecular dynamic simulations were conducted to assess the stability of retinal-protein interactions.

## 2. Materials and Methods

### 2.1. Expression and Purification of Bacteriorhodopsin Proteins

The nucleic acid genome was extracted from *Halorubrum* sp. ejinoor. PCR amplification was then performed using *He*AR-specific primers. The PCR product was separated by agarose gel electrophoresis and cloned into the pT7blue plasmid (Novagen/Merck, Darmstadt, Germany). The obtained DNA sequence was analyzed using an ABI3130/3130xl genetic analyzer (Applied Biosystems, Foster City, CA, USA) [23]. The *He*AR gene was digested with NdeI/XhoI enzymes (New England Biolabs, Ipswich, MA, USA) and inserted into the pET21c(+) plasmid (Novagen/Merck, Darmstadt, Germany) to express *He*AR. This insertion strategy resulted in an additional 8 amino acids (LEHHHHHH) at the C-terminus, which makes subsequent purification convenient. The PCR-amplified *He*AR gene was first cloned into the pT7blue plasmid for TA cloning and sequencing to verify insert orientation and sequence integrity prior to subcloning into the expression vector pET21c(+). The inserted gene sequence was confirmed by sequencing again using an ABI3130/3130xl genetic analyzer. The recombinant plasmid was then transformed into *Escherichia coli* BL21 (DE3) strain (Invitrogen, Carlsbad, CA, USA) for expression [24].

*Escherichia coli* BL21 (DE3) cells were grown in lysogeny broth (LB) medium supplemented with 100 μg/mL ampicillin at 37 °C with agitation (180 rpm) until the optical density at 600 nm (OD600) reached 0.6, corresponding to the mid-exponential growth phase. This strain was selected for its demonstrated capacity to express membrane proteins at high yields and compatibility with the pET21c(+) expression vector. To induce target protein expression, cultures were supplemented with 1 mM isopropyl β-D-1-thiogalactopyranoside (IPTG) and 10 μM All *trans* retinal, followed by incubation at 25 °C for 8 h under continuous shaking (180 rpm). This low-temperature induction protocol was adopted to mitigate inclusion body formation and enhance soluble protein recovery [25].

After induction for 8 h, cells were harvested by centrifugation at 6000× *g* for 30 min. The cell pellet was then lysed and membrane proteins were solubilized in buffer containing 1.5% n-dodecyl-β-D-maltopyranoside (DDM) as a detergent [26]. Purification of *He*AR and BR was further carried out using Ni-NTA agarose chromatography to separate the membrane protein from the *Escherichia coli* BL21 (DE3) strain [27]. *Hs* was cultured in *halobacterial* medium, and BR was purified by sucrose density gradient ultracentrifugation. The purified *He*AR and BR were subjected to biological characterization.

### 2.2. Size-Exclusion Chromatography (SEC) Analysis and CD Spectra

Size-exclusion chromatographic separations were conducted under ambient temperature conditions (25 ± 1 °C) utilizing a Superdex™ 200 10/300 GL column (Cytiva, Marlborough, MA, USA) pre-calibrated with a high-molecular-weight calibration kit. The chromatographic system comprised an ÄKTA Purifier™ FPLC platform (Cytiva) equipped with a 200 μL sample injection loop. Molecular weight standards included ferritin (440 kDa), aldolase (158 kDa), conalbumin (75 kDa), and ovalbumin (43 kDa). Column equilibration was achieved by continuous perfusion with 1.5 column volumes of a physiological buffer system containing 10 mM HEPES (4-(2-hydroxyethyl)-1-piperazineethanesulfonic acid) buffer (pH 7.5), 100 mM NaCl, and 0.05% (*w*/*v*) n-dodecyl-β-D-maltoside (DDM) to maintain protein monodispersity [28]. Purified *He*AR samples underwent ultracentrifugation at 100,000× *g* for 45 min (4 °C) to eliminate particulate aggregates prior to loading. Chromatographic separation proceeded isocratically at a flow rate of 0.5 mL/min, with UV-Vis detection at 280 nm (aromatic residues) and 560 nm (retinal chromophore). Elution profiles were monitored in real-time using UNICORN™ 7.0 software. Protein-containing fractions corresponding to the dominant absorbance peaks were collected for subsequent biophysical characterization. Final protein concentrations ranged from 0.1–0.3 mg/mL. Circular dichroism (CD) spectra of *He*AR and BR were recorded in a solution containing 0.05% DDM and 10 mM HEPES (pH 7.5) using a JASCO J-1500 CD spectrometer (JASCO Corporation, Tokyo, Japan). HEPES buffer (pH 7.5) was chosen to mimic physiological conditions, while DDM (0.05%) was used to solubilize membrane proteins without destabilizing its trimeric structure, as confirmed by prior stability assays [28]. HEPES (pH 7.5) was chosen to mimic physiological conditions, while DDM (0.05%) stabilized transmembrane domains without disrupting trimerization, as confirmed by SEC and CD stability assays.

### 2.3. pH Titration and Absorption Spectra

The apparent pKa value of the protonated Schiff base counterion was determined by monitoring pH-dependent spectral shifts at λmax = 620 nm using a hexa-buffered system (citric acid/MES/HEPES/MOPS/CHES/CAPS, each 10 mM) spanning pH 1.0–9.0. This multi-component buffer strategy maintained constant ionic strength across the full pH range while minimizing buffer-specific artifacts. *He*AR and BR were dissolved in a 10 mM HEPES buffer (4-(2-Hydroxyethyl)-1-piperazineethanesulfonic acid, pH 7.5) containing 0.05% DDM and were left at room temperature overnight to create dark-adapted samples, HeAR^D^ and BR^D^ [29]. After illuminating the *He*AR^D^ and BR^D^ solutions with 500 nm light to obtain the light-adapted samples, *He*AR^L^ and BR^L^, the absorption spectra of both light- and dark-adapted samples were measured using an MPS2000 UV-visible spectrophotometer (Shimadzu Corporation, Kyoto, Japan).

### 2.4. Analysis by High Performance Liquid Chromatography of Isomeric Yellow Dye

The extraction method for the retinylidene photoreceptor and bacteriorhodopsin (*He*AR and BR) followed the protocol established by Dai et al. [30]. The isomeric content of retinylidene chromophores in both light and dark-adapted states was analyzed using high-performance liquid chromatography with a JASCO FLC-350 (JASCO Corporation, Tokyo, Japan). A total of 100 μL of *He*AR and BR (0.05% DDM, 10 mM HEPES, pH 7.5) was extracted. Then, 100 μL of hydroxylamine and 300 μL of methanol were added and mixed thoroughly, followed by the addition of 600 μL of hexane. After centrifugation, the supernatant was collected, and the organic solvent was dried under nitrogen. Finally, 50 μL of hexane was added for high-performance liquid chromatography detection.

### 2.5. Flash-Photolysis Analysis

Light-induced absorbance changes were measured using an ND-YAG laser (Spectra-Physics, Santa Clara, CA, USA) (532 nm, 7 ns) flash photolysis setup [31]. Absorbance changes were measured at 10 nm intervals across the visible range (320–700 nm). The flash photolysis data analysis referenced the model developed by Dai et al. [30]. Three independent replicates were performed for flash photolysis assays (*p* < 0.05). All samples were prepared in a solution containing 10 mM HEPES, 100 mM NaCl, 0.05% DDM, pH 7.5, with an optical density (OD560) of 0.7.

### 2.6. Proton Translocation Kinetics and Intracellular ATP Quantification

Proton translocation activities in intact cells *Escherichia coli* BL21 (DE3) expressing *He*AR suspensions were monitored using a Thermo Scientific™ Orion™ micro-pH electrode (Thermo Scientific, Waltham, MA, USA) under actinic illumination (λ = 590 ± 8.5 nm bandwidth, irradiance 10 mW·cm^−2^). Experiments were conducted in a thermostatically controlled quartz cuvette (25.0 ± 0.2 °C) containing cells resuspended in carbonate-free physiological buffer (150 mM NaCl, 10 mM HEPES [4-(2-hydroxyethyl)-1-piperazineethanesulfonic acid], pH 7.5) at OD_600_ = 1.0 ± 0.05. Electrode calibration was performed using NIST-traceable pH standards (pH 4.01 ± 0.02 and 7.00 ± 0.02). Proton motive force (PMF) dependency was investigated through pretreatment with 10 μM carbonyl cyanide *m*-chlorophenylhydrazone (CCCP), a protonophore enabling H⁺-gradient dissipation. For ATP synthase inhibition studies, cells were preincubated with 50 μM *N*,*N′*-dicyclohexylcarbodiimide (DCCD) in dark-adapted conditions (30 min, 37 °C) following established protocols. The luciferine-luciferase assay, as outlined in a previous study, was employed to measure the ATP levels in whole cells [32].

### 2.7. Computational Characterization of HeAR

The tertiary structures of *He*AR and BR were predicted using AlphaFold https://www.alphafold.ebi.ac.uk (accessed on 3 March 2025). through multiple sequence alignment, with conformation selection based on predicted local distance difference test (pLDDT) confidence thresholds (>90) [33]. Structural alignments against homologs Archaerhodopsin 3 (AR3) (PDB:6GUX) and BR (PDB:1C3W) were executed in ChimeraX, revealing conserved retinal-binding domain architecture [34]. Molecular docking simulations employed All-*trans* retinal chromophores (retrieved from the TCMSP database), parameterized using the OpenBael toolkit v3.0.0. Semi-flexible docking procedures were executed using AutoDock Vina v1.2.3 within a cubic search space, yielding ten predominant pose clusters optimized through iterative energy minimization. Binding pose selection employed a thermodynamic threshold of ΔG ≤ −5.0 kcal·mol^−1^, corresponding to physiologically relevant ligand-receptor interactions. Spatiotemporal mapping of hydrogen-bond networks was performed through PyMOL v2.5.4-based occupancy probability analysis [35]. Resultant ligand-binding conformations were cross-validated against experimentally resolved archaeal rhodopsin architectures (AR1: PDB 1UAZ; AR2: PDB 1VGO) and the BR (PDB 1C3W), ensuring evolutionary consistency in retinal-binding pocket organization. Molecular dynamics simulations implemented AMBER GAFF2 ligand parameters and OPLS-AA/M protein force field within a rhombic dodecahedron solvation box (TIP3P water model, ≥1.2 nm buffer), with charge neutralization achieved through 0.15 M NaCl addition. Energy minimization (50,000 steepest descent steps, 10 kJ·mol^−1^·nm^−1^ convergence) preceded sequential NVT (100 ps, 310 K) and NPT (100 ps, 1 bar) equilibration phases. Production simulations (100 ns duration, 2 fs timestep) employed LINCS-constrained bonds under periodic boundary conditions, with trajectory analyses (RMSD, the number of hydrogen bonds, the radius of gyration and total energy) conducted via GROMACS routines [36].

## 3. Results

### 3.1. Induction Expression and Purification of Photosensitive Proteins

**Figure 1:**
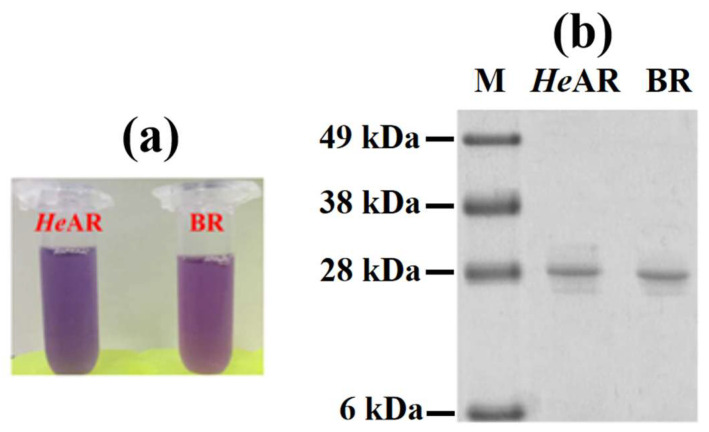
Microbial rhodopsins: BR and *He*AR. (**a**) Comparison of the color of the *He*AR and BR. (**b**) The expression of the *He*AR and BR analyzed by SDS-PAGE.

The *He*AR gene was cloned into the pET21c(+) vector and transformed into *E. coli* BL21 (DE3) for protein expression. Following an 8 h induction with IPTG, the bacterial culture transitioned from pale yellow to a deep purple-red. The purified *He*AR and BR proteins exhibited a purple color (Figure 1a), indicating that bacteria with active proton pumps might appear purple because of these proteins. SDS-PAGE analysis of *He*AR and BR showed distinct bands at approximately 27 kDa (Figure 1b). This finding aligns with the predicted molecular weights derived from the amino acid sequences of both proteins cloned into the vector, confirming successful purification.

### 3.2. SEC Elution Profile and Secondary Structure Characterization

Size-exclusion chromatography (SEC) analysis elucidated the oligomeric states and detergent-binding properties of bacteriorhodopsin (BR) and its homolog *He*AR. The intrinsic hydrophobicity of transmembrane domains—enriched with leucine, isoleucine, and phenylalanine residues—dictates their instability in aqueous environments. Mechanistic studies on detergent-mediated membrane protein extraction revealed that dodecyl maltoside (DDM) molecules, at concentrations exceeding the critical micellar concentration, stabilize the hydrophobic transmembrane regions via their alkyl tails while forming hydrophilic interfaces with the aqueous phase through polar headgroups, thereby generating monodisperse “protein-lipid-detergent ternary complexes”. Conversely, suboptimal detergent concentrations expose hydrophobic surfaces, thereby inducing irreversible aggregation or conformational alterations [28].

**Figure 2:**
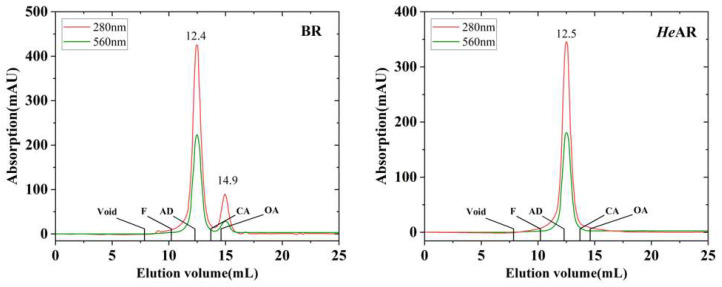
Trimeric Assembly Conservation Revealed by SEC Analysis of *He*AR and BR. Elution profiles of *He*AR and BR were acquired using a Superdex 200 10/300 GL chromatography system, with eluent absorbance monitored at 280 nm (red trace) and 560 nm (green trace). Annotated retention volumes correspond to characteristic peaks of *He*AR and BR (560 nm-specific absorbance), alongside void volume markers and molecular weight standards: thyroglobulin (TG, 669 kDa), aldolase (AD, 158 kDa), conalbumin (CA, 75 kDa), and ovalbumin (OA, 43 kDa).

SEC elution profiles (Figure 2) revealed a bimodal distribution for BR under 280 nm detection: a dominant peak at 12.4 mL (131 kDa) and a minor peak at 14.9 mL (41.2 kDa). Specific 560 nm detection confirmed that both peaks corresponded to intact pigment-binding proteins. Notably, the apparent molecular weight of the dominant peak (131 kDa) significantly exceeded the 27 kDa monomeric mass determined by SDS-PAGE, which aligned with the sequence-predicted value of 28.2 kDa. A trimer model rationalized this discrepancy: the protein core (3 × 28.2 kDa = 84.6 kDa) accounted for 64.5% of the observed mass (131 kDa), with the remaining 46.4 kDa attributed to DDM binding. Assuming a DDM molecular weight of 0.5 kDa, approximately 93 detergent molecules associate with each BR trimer. This calculation aligns with reported characteristics of transmembrane protein-detergent complexes, confirming BR’s native trimeric architecture. SEC analysis of *He*AR yielded a single elution peak at 12.5 mL (129 kDa), closely mirroring BR’s trimeric elution profile (12.4 mL, 131 kDa). Homology-based trimer modeling indicated a protein core mass of 83.1 kDa (3 × 27.7 kDa), leaving a 45.9 kDa mass differential attributable to ~92 bound DDM molecules. This finding substantiates that *He*AR, recombinantly expressed in *E. coli* BL21 (DE3), spontaneously adopts a three-dimensional assembly analogous to native BR.

**Figure 3:**
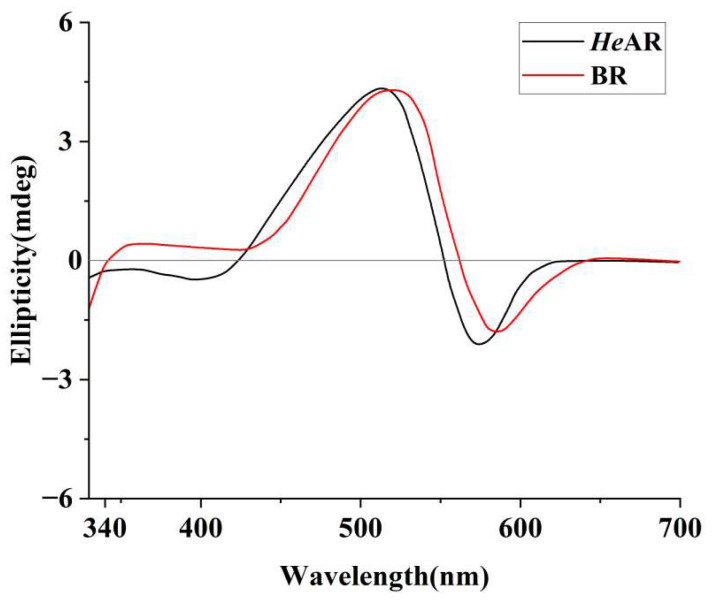
Circular dichroism (CD) spectra of *He*AR and BR. CD spectra were recorded in 10 mM HEPES buffer (pH 7.5) containing 0.05% DDM at 25 °C. Both *He*AR (black line) and BR (red line) exhibit characteristic positive peaks near 525–535 nm and negative peaks near 567–577 nm.

Circular dichroism (CD) analysis under near-physiological conditions (pH 7.5, 10 mM HEPES, 0.05% DDM) revealed structural congruence between *He*AR and BR, as evidenced by analogous spectral profiles (Figure 3). Notably, *He*AR exhibited a 10 nm red-shift in its maximum absorption wavelength relative to BR, with positive ellipticity peaks at 525 nm (*He*AR) and 535 nm (BR) and negative troughs at 567 nm (*He*AR) and 577 nm (BR). This spectral divergence implies localized perturbations in the chromophore microenvironment, potentially arising from altered tryptophan residue orientations or helical packing geometries within the trimeric assembly. Critically, the conserved biphasic CD signatures—with hallmarks of retinal-binding pocket rigidity and interprotomer π-π stacking—corroborate SEC-derived conclusions regarding shared trimeric architectures.

### 3.3. Light and Dark Adaptation States and Composition of Visual Arrestin Isoforms

**Figure 4:**
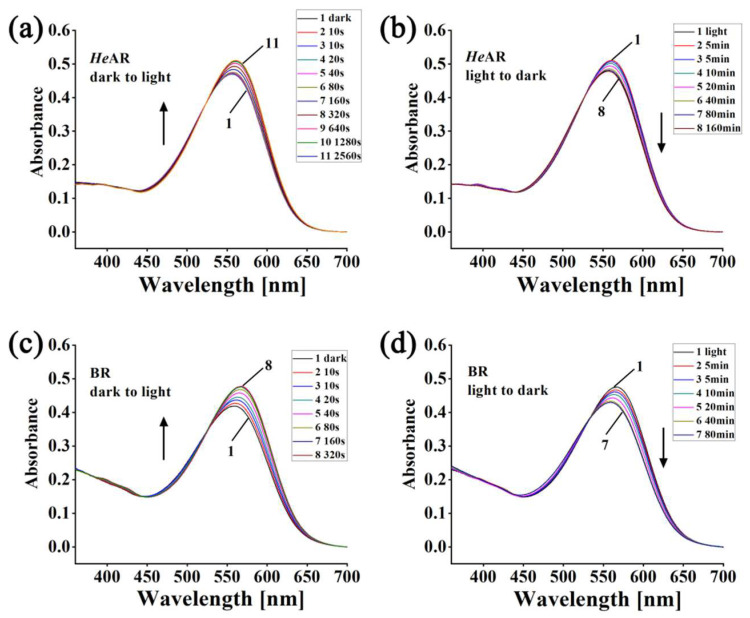
Light–dark adaptation dynamics of purified *He*AR and BR in 10 mM HEPES (pH 7.5) at 20 °C. (**a**) Light adaptation of *He*AR. Curve 1: Dark-adapted *He*AR. Curves 2–11: Spectral evolution during sequential 500 nm irradiation (10, 10, 20, 40, 80, 160, 320, 640, 1280, and 2560 s). (**b**) Dark adaptation of *He*AR. Curve 1: Light-adapted *He*AR. Curves 2–8: Spectral recovery during dark incubation measured at 5, 5, 10, 20, 40, 80, and 160 min. (**c**) Light adaptation of BR. Curve 1: Dark-adapted BR. Curves 2–8: Spectral changes under sequential 500 nm irradiation (10, 10, 20, 40, 80, 160, and 320 s). (**d**) Dark adaptation of BR. Curve 1: Light-adapted BR. Curves 2–7: Spectral recovery during dark incubation measured at 5, 5, 10, 20, 40, and 80 min. The arrow indicates the changes in absorption spectra from Curve 1 to Curve n.

Bacteriorhodopsin (BR) interacts with the retinal chromophore in its binding region, allowing it to absorb a wide range of wavelengths in the visible spectrum and to exhibit both light and dark adaptation states, with maximum absorption peak wavelengths (λmax) of 578 nm for light adaptation and 568 nm for dark adaptation. In darkness, both the All-*trans* and 13-*cis* forms of retinal coexist. When illuminated, the 13-*cis* retinal chromophore converts to the All-*trans* form, leading to the light-adapted state. If the light-adapted protein is placed in darkness, it reverts to the dark-adapted state. *He*AR also shows light and dark adaptation states. The transformation from *He*AR^D^ with a maximum absorption of 550 nm to *He*AR^L^ with a maximum absorption of 560 nm takes 2560 s (Figure 4a). The transition from BR^D^ (λmax 568) to BR^L^ (λmax 578 nm) requires 320 s, with a 10 nm blue shift in both cases (Figure 4c). Figure 4b shows that *He*AR^L^ changes to *He*AR^D^ after about 160 min in the dark, whereas BR^L^ converts to BR^D^ in 80 min (Figure 4d). Variations in the composition of retinal chromophore isomers in proteins can cause changes in their absorption spectrum maximum values.

**Figure 5:**
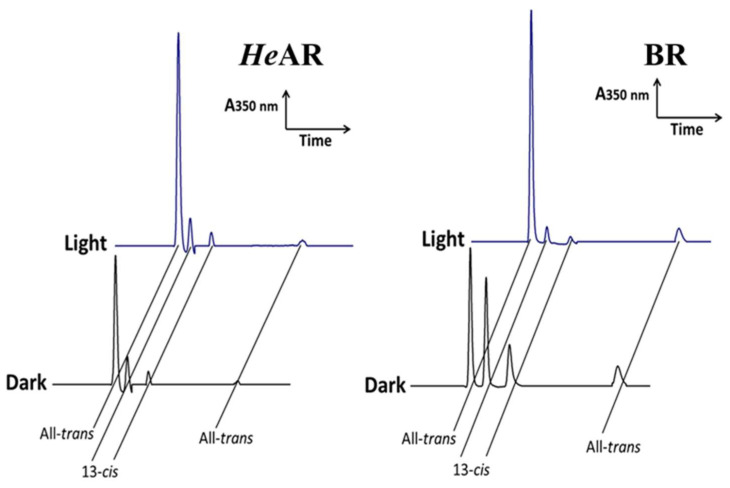
Retinal Isomer Composition Analysis of Light/Dark-Adapted *He*AR and BR by HPLC. Chromatographic separation was performed on a JASCO FLC-350 system equipped with a C18 reverse-phase column (5 μm, 4.6 × 250 mm) using isocratic elution (hexane:isopropanol = 95:5, 1.0 mL/min). Retinal isomers were detected at 350 nm (retinylidene chromophore absorption) after extraction with hydroxylamine/methanol/hexane.

To determine the isomeric composition of retinal chromophores in both light and dark adaptation states of *He*AR, we analyzed the extracted retinal chromophores using high performance liquid chromatography (HPLC). Retinal chromophores exhibit a strong absorption peak at 350 nm, allowing us to calculate their content ratios from the peak values. HPLC analysis showed that both *He*AR and BR contain only two retinal chromophore isomers: All-*trans* and 13-*cis*. The calculated ratios of retinal isomers are as follows: *He*AR^D^ has a ratio of All-*trans* to 13-*cis* of 2:1, *He*AR^L^ has 6:1, BR^D^ has 1:1, and BR^L^ has 6:1 (Figure 5). The results obtained for BR are consistent with those reported by Maeda et al. [37] previously. This indicates that during the transformation of *He*AR from light to dark adaptation states, as the content of 13-*cis* retinal chromophore decreases, the content of All-*trans* retinal chromophore gradually increases. It is possible that, during the photochemical cycle reaction, the isomeric composition of retinal chromophores undergoes changes from All-*trans* to 13-*cis* and back to All-*trans*.

**Figure 6:**
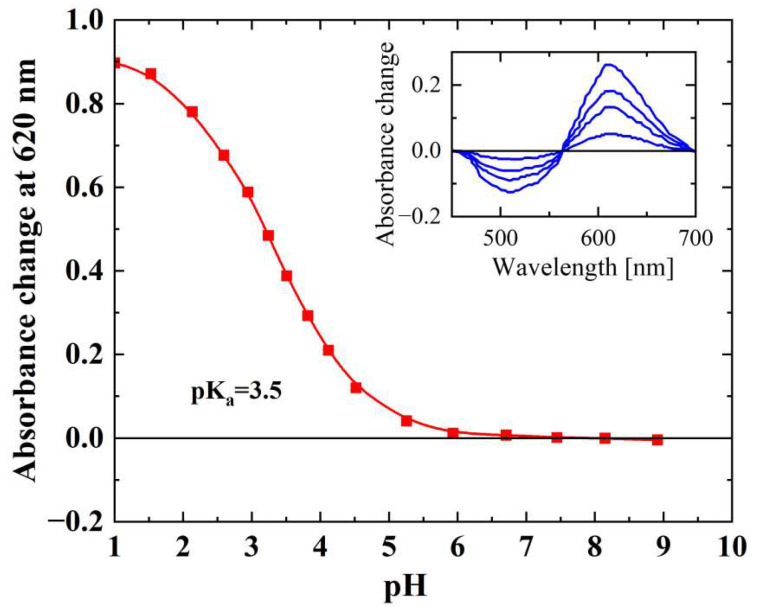
pH-dependent spectral evolution and quantitative analysis of Asp-95 proton affinity in *He*AR. Steady-state pH-dependent spectroscopic titration resolved the protonation dynamics of Asp-95, the counterion to the protonated Schiff base in *He*AR. Systematic acidification induced a red-shift of the retinal absorption maximum from 550 nm to 620 nm (spectral transition trajectory shown in inset), originating from polarization redistribution within the retinal conjugated system following Asp-95 carboxyl group protonation. Nonlinear regression analysis of absorbance variations at 620 nm yielded a proton dissociation constant (pKa = 3.5 ± 0.1) for Asp-95.

Under acidic conditions, *He*AR displayed a characteristic red-shift in absorption maximum to 620 nm. Quantitative analysis of this spectral transition using the Henderson–Hasselbalch equation revealed a proton dissociation constant (pKa = 3.5 ± 0.1) for Asp-95, which serves as the counterion to the protonated Schiff base and corresponds structurally to Asp-85 in BR (Figure 6). The observed pKa elevation—relative to the value of ~2.6 for Asp-85 in BR—implies evolutionary divergence in electrostatic microenvironment regulation within the retinal-binding pocket [38].

### 3.4. Photosensitive Protein Photochemical Reaction Cycle

**Figure 7:**
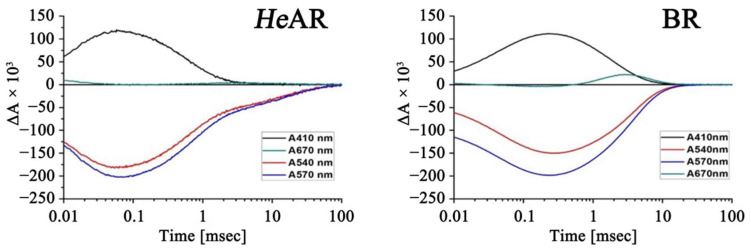
Comparative Kinetics of Flash-Induced Absorbance Changes in *He*AR and BR. Time-resolved absorbance dynamics were recorded at four wavelengths (410, 540, 570, and 670 nm) following 532 nm laser excitation (7 ns pulse width, 10 mJ/cm^2^ fluence) in 10 mM HEPES buffer (pH 7.5, 0.05% DDM, 25 °C). Data represent triplicate measurements (*p* < 0.05).

After absorbing light, BR undergoes a photoreaction cycle. Upon light exposure, BR rapidly forms various structural intermediates. Each intermediate has unique maximum absorption peaks. In this study, using BR as a model, the time-dependent absorption wavelength changes intermediates that represent different states, namely M (410 nm), L/N (540 nm), O (670 nm), and the ground state (570 nm), were analyzed. As shown in Figure 7, *He*AR also exhibits L, M, and O intermediates similar to BR, which change rapidly over a very short period, transitioning between states before returning to the ground state. Initially, the signals for both the *He*AR ground state and L state decrease at 60 µs, while the M state signal increases and reaches saturation at the same time. Subsequently, as the M state signal weakens, a faint O state signal increases, with the M state signal disappearing completely at 3.3 ms, taking a total of 100 ms for all intermediates to vanish. Similarly, for BR, the L state and ground state signals decrease (0.3 ms), while the M state signal increases, reaching saturation at 0.2 ms. Shortly after, the M state signal decreases while the O state signal increases, followed by a return to the ground state, completing the entire cycle in about 11 ms. These results indicate that *He*AR also undergoes a photoreaction cycle, but with a longer duration compared to BR. Notably, the generation and disappearance times of the M state in *He*AR are quicker than in BR, as the M state disappears before the O state does in *He*AR. In contrast, in BR, the M state and O state return to the ground state together. This suggests that *He*AR may involve additional intermediates that contribute to the slow cycling process. However, *He*AR displayed accelerated M-state decay kinetics (saturation at 60 µs, full decay by 3.3 ms) compared to BR (M-state saturation at 0.2 ms, decay synchronizing with O-state at 11 ms). This decoupling of M- and O-state lifetimes in *He*AR (total cycle duration: 100 ms vs. BR’s 11 ms) suggests a bifurcated relaxation pathway, potentially involving unobserved late-M substates or alternative proton transfer routes [39]. *He*AR exhibits a photocycle duration of 100 ms, positioning it between the rapid kinetics of bacteriorhodopsin (BR, 11 ms) and the prolonged cycling of xanthorhodopsin (250 ms). Notably, *He*AR’s kinetics align more closely with *Halorubrum* homologs AR1/AR2 (85 ms) than with phylogenetically distant rhodopsins [40,41], suggesting genus-specific evolutionary constraints on proton-pumping efficiency.

### 3.5. Light-Driven Proton-ATP Metabolic Coupling Dynamics

**Figure 8:**
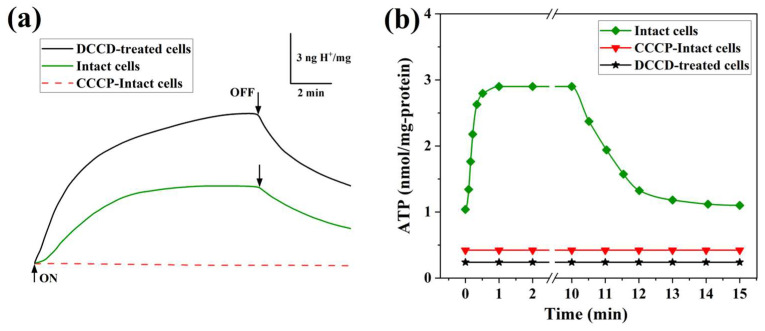
Real-time kinetic profiling of photon-driven proton flux coupling with ATP biosynthetic efficiency. (**a**) Light-induced dynamic pH response profiles in *He*AR-expressing *E. coli* BL21 (DE3) cell suspensions, demonstrating real-time monitoring of transmembrane proton gradient dynamics mediated by *He*AR’s photosensitive properties. (**b**) Quantitative analysis of light-activated intracellular ATP biosynthesis in *He*AR-engineered *E. coli* BL21 (DE3) populations.

BR has been conclusively demonstrated to function as a light-driven proton pump, establishing transmembrane electrical potentials within milliseconds of illumination to power H⁺-ATPase activity [42]. This rapid photoelectric response enables direct coupling between photon capture and ATP biosynthesis, suggesting evolutionary optimization for energy harvesting efficiency in dynamic light environments. Building upon prior investigations utilizing the engineered strain *Hb. salinarium* Pho81BR (ΔhR/ΔsR-I/ΔsR-II), which established H⁺-ATPase-mediated gated proton uptake through DCCD-sensitive mechanisms [32], we employed DCCD to dissect compensatory proton translocation dynamics in the current study. Proton pump activity analysis of *He*AR-expressing *E. coli* BL21 (DE3) suspensions revealed light-dependent acidification kinetics achieving steady-state within 4 min (Figure 8a), with quantifiable proton extrusion at 0.1 ng H⁺/mg protein·s. Strikingly, 50 μM DCCD treatment potentiated both the rate (0.3 ng H⁺/mg protein·s) and amplitude of photoinduced proton efflux, indicative of *He*AR-mediated compensatory flux amplification following H⁺-ATPase inhibition. Post-illumination pH restitution kinetics exhibited accelerated proton influx in DCCD-treated systems (Figure 8a), aligning with disrupted ATPase-dependent dark-state proton homeostasis. Complete eradication of photoresponsive pH oscillations by 10 μM CCCP validated strict reliance on preserved chemiosmotic gradients rather than direct photochemical coupling. Parallel ATP flux quantification unveiled ultrafast photophosphorylation kinetics, with cellular ATP reservoirs attaining steady-state within 30 s of illumination (0.3 nmol ATP/mg proteins; Figure 8b)—a temporal resolution outperforming canonical photosynthetic frameworks by two orders of magnitude, underscoring archaeal rhodopsin specialization for transient energy transduction. Conserved ATP synthesis rates across *Halobacterium halobium* (RIM1 and R1) control strains emphasized phylogenetic preservation of this bioenergetic paradigm [43]. Photon withdrawal precipitated rapid ATP depletion (hydrolysis rate: 0.02 nmol/mg protein·s), restoring dark-anaerobic equilibrium within 3 min. Synergistic application of 50 mM DCCD and 10 μM CCCP not only diminished basal ATP reserves but abolished light-driven synthesis entirely (Figure 8b), unequivocally establishing dual dependency on both proton-motive force genesis and ATPase catalytic competence. These findings holistically delineate *He*AR’s unique capacity to interface with conserved energy transduction machinery while exhibiting adaptive regulatory plasticity—a duality rendering it particularly amenable to optobioenergetic engineering applications. This suggests that, like BR, *He*AR possesses the ability to transport protons outward. Proton pump activity is a crucial factor in achieving light-controlled target cell modulation as it can induce membrane hyperpolarization and inhibit cell excitability. Currently, light-driven proton pumps, such as AR3, are used to efficiently optogenetically silence neuron cells. Under yellow light activation, proton-pumping proteins move positively charged protons from inside neurons to the external environment. This process leads to hyperpolarization, which helps maintain neuron stability. When the yellow light is turned off, AR3 rapidly closes ion transport channels compared to *Np*HR, enabling quick light control over neuron cells at low light power. Therefore, light-driven proton pumps are considered highly efficient tools for optogenetic applications. *He*AR, as a proton transporter, may also serve as an excellent photosensitive tool for optically inhibiting neuron cells.

### 3.6. Phylogenetic Tree and Amino Acid Conservation Profiling of Archaerhodopsins

**Figure 9:**
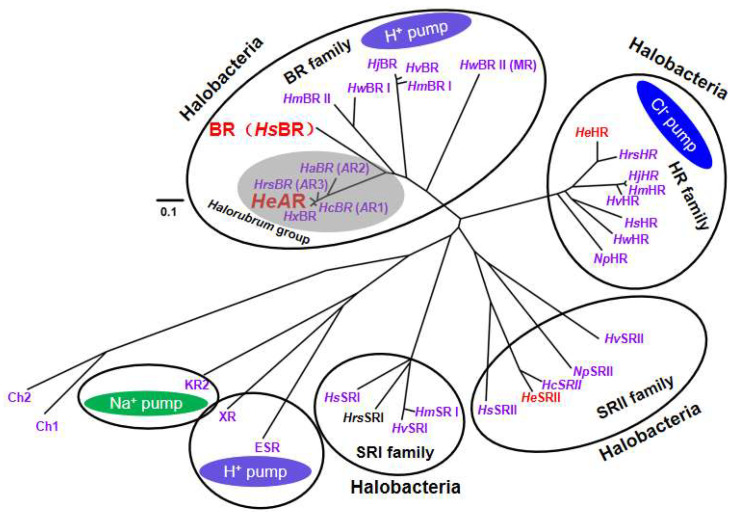
Phylogenetic tree of microbial rhodopsins. *Halorubrum* sp. ejinoor (*He*)rhodopsins (*He*AR, *He*HR and *He*SRII), can be classified to their respective archaeal rhodopsin groups. ‘*Hc*’, ‘*Ha*’, ‘*Hrs*’, ‘*Hx*’, ‘*Hs*’, ‘*Hw* I(and *Hw* II)’, ‘*Hm* I(and *Hm* II)’, ‘*Hj*’, ‘*Hv*’, and ‘*Np*’ represent microbial rhodopsins from *Halorubrum chaoviator*, *Halorubrum* sp. aus 2, *Halorubrum sodomense*, *Halorubrum xinjiangense*, *Halobacterium salinarum*, *Haloquadratum walsbyi*, *Haloarcula marismortui*, *Haloarcula japonica*, *Haloarcula vallismortis*, and *Natronomonas pharaonis*, respectively. Green represents sodium ion pumps, purple represents proton pumps, blue represents chloride ion pumps, and the gray section indicates proton pumps specific to the genus *Halorubrum*. The scale bar represents 0.1 expected changes per site.

Phylogenetic analysis of amino acid sequences (Figure 9) confirmed that *He*AR belongs to the archaeal proton pump family within the genus *Halorubrum* (Halobacteriaceae), exhibiting the highest sequence homology (95%) with *Hx*BR (Archaerhodopsin AR4) derived from *Halorubrum xinjiangense* [44]. The 16S rRNA phylogenetic lineage of this strain showed 99.0% similarity to *Halorubrum* sp. Ejinoor isolated from a salt lake in Inner Mongolia, with both strains originating from hypersaline environments in Xinjiang and Inner Mongolia, China. Notably, the genome of *Halorubrum* sp. Ejinoor encodes three rhodopsin homologs (*He*AR, *He*HR, and *He*SRII), whereas *Halorubrum xinjiangense* has thus far been reported to express only AR4-class proteins, suggesting strain-specific evolutionary trajectories in phototrophic metabolic adaptation. *He*AR demonstrated 93% transmembrane domain homology with AR3 from *Halorubrum sodomense*, along with graded homology to AR1 (94%) and AR2 (86%) from *Halorubrum chaoviator* and *Halorubrum* sp. aus 2, respectively, significantly surpassing its conservation with bacterial rhodopsin BR (57%) [45].

**Figure 10:**
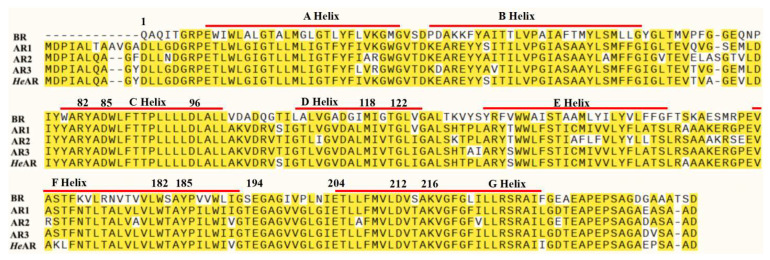
Comparative amino acid sequence alignment between Bacteriorhodopsin (BR), Archaerhodopsins (AR1, AR2, AR3) and *He*AR. Proton transport amino acids with numbering and helical segment (uplined) for BR.

Systematic comparisons revealed that Archaerhodopsins (ARs) within the *Halorubrum* genus, despite substantial sequence divergence from BR, display strict evolutionary conservation of 11 critical residues associated with proton-pumping functionality—including the Schiff base-binding site and proton release/uptake channel residues—while exhibiting convergent photochemical cycling properties with BR. Recent studies have highlighted AR3 as a pivotal molecular tool in optogenetics due to its unique light-gated membrane voltage responsiveness, enabling precise neuromodulation in neural circuits [46,47]. Our multidimensional structural alignment revealed a highly conserved stereochemical network within the proton-translocating core regions (C and G helices) of *He*AR, AR3, and BR: Eleven functional residues in *He*AR (R92, D95, D106, M128, and seven others) exhibited strict spatial correspondence with their BR counterparts (R82, D85, D96, M118, etc.; Figure 10). Intriguingly, the topological congruence at the proton release cluster and cytoplasmic channel entrance further elucidates evolutionarily conserved mechanisms underlying cross-species proton pump functionality.

### 3.7. AlphaFold Predicts Functional Microbial Rhodopsin Structures

**Figure 11:**
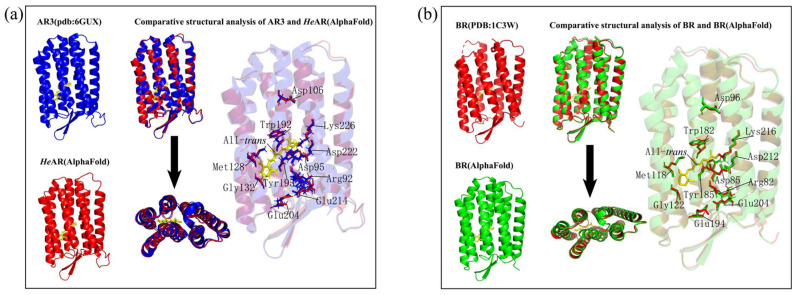
Structural congruence between AlphaFold-predicted models and experimental archaeal/bacterial rhodopsin architectures. (**a**) Superimposed topology of the AlphaFold-derived *He*AR (red ribbon) with the X-ray crystallographic AR3 structure (PDB: 6GUX, blue surface) demonstrates conserved seven-transmembrane helical packing (Cα-RMSD = 1.3 Å); (**b**) Atomic-level alignment of the computational BR model (green cartoon) against its experimental counterpart (PDB: 1C3W, magenta sticks) reveals sub-Ångström precision in helical core geometry (Cα-RMSD = 0.8 Å). All alignments were refined through iterative Kabsch optimization (PyMOL).

Leveraging the deep learning-driven AlphaFold prediction framework, we generated full-atom tertiary structural models of the *He*AR and bacteriorhodopsin (BR) (Figure 11). Both models revealed the canonical seven-transmembrane α-helical topology (TM1-TM7), with their global folding architectures exhibiting remarkable congruence to experimentally resolved crystal structures of AR3 (PDB:6GUX) [48] and BR (PDB:1C3W) [49], as evidenced by topology consistency indices (TM-scores) exceeding 0.92. Rigorous transmembrane helix alignment analysis demonstrated exceptional atomic-level precision: the Cα root mean square deviation (RMSD) between *He*AR’s TM1-TM7 helical bundle and its counterpart in AR3 measured 1.3 Å, while BR’s corresponding helices aligned with the 1C3W structure at an ultrahigh precision of 0.8 Å RMSD, unequivocally validating the physiological plausibility of these computational models. Strikingly, 11 evolutionarily conserved residues constituting the proton translocation pathway—including the Schiff base linkage site and critical proton release/uptake switch residues—were precisely localized within three-dimensional hydrophobic cavities of the transmembrane channel. These functional determinants exhibited spatial coordinate deviations of <15° in side-chain orientation relative to their crystallographically resolved counterparts, a finding that not only corroborates the geometric fidelity of AlphaFold predictions but also underscores its unprecedented capability to resolve dynamic functional microenvironments. Comparative analysis of proton wire architecture revealed that key hydrogen-bonding distances within the *He*AR proton channel deviated by merely 0.2–0.3 Å from AR3’s experimental values. These multi-scale validations collectively establish AlphaFold-derived models as not merely structural approximations, but rather as predictive tools capable of recapitulating functional features integral to microbial rhodopsin physiology.

### 3.8. Molecular Docking Analysis of HeAR and BR with All-Trans Retinal Using Alphafold-Predicted Structures

**Figure 12:**
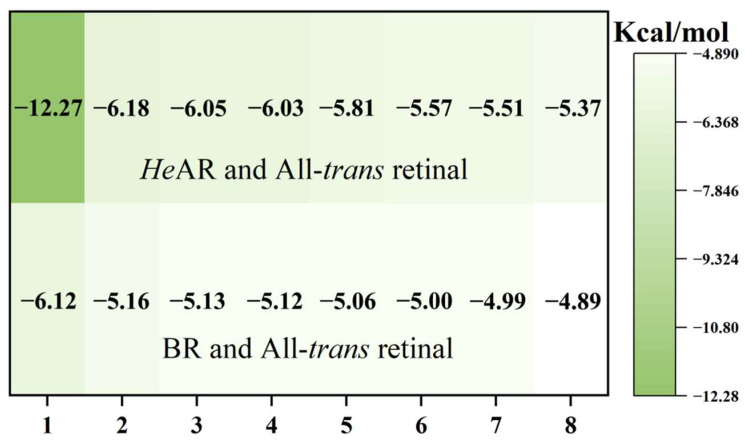
Binding energies were calculated using molecular docking simulations with AlphaFold-predicted structures of *He*AR and BR. All-*trans* retinal was used as the ligand. The energy values are expressed in kcal/mol. Lower binding energy indicates stronger interaction between the protein and the ligand.

**Figure 13:**
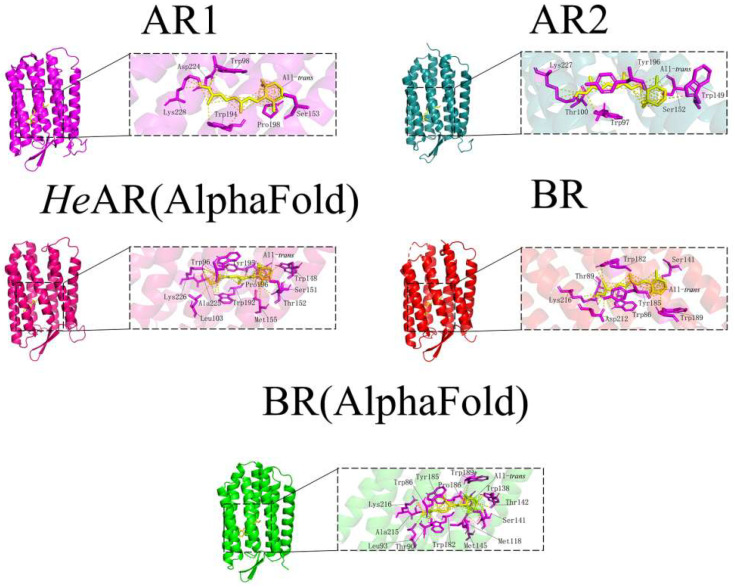
Molecular docking analysis of All-*trans* retinal with *He*AR, ARs, and BR proteins. Visualization of the molecular docking between All-*trans* retinal and the predicted structures of *He*AR and BR (generated by AlphaFold), as well as known structures of ARs (PDB: 1UAZ for AR1; PDB: 1VGO for AR2) and BR (PDB: 1C3W). The docking interactions were constrained to a 3.5 Å radius.

This study systematically investigated the molecular interactions between All-*trans* retinal (ATR) and bacteriorhodopsin-family proteins through a robust molecular docking approach. The experimental framework encompassed Alphafold-predicted models of *He*AR and BR, alongside crystallographically resolved structures of AR1 (PDB 1UAZ) [50], AR2 (PDB 1VGO) [51], and BR (PDB 1C3W). Each receptor-ligand complex underwent 10 independent docking simulations to ensure statistical robustness, with binding free energy (ΔG) calculated via the MM/PBSA method. Structural analyses were augmented by PyMOL for three-dimensional visualization and Origin 2021 for thermodynamic profiling. Quantitative analysis revealed pronounced binding affinities of ATR for both predicted *He*AR and BR models, with all ΔG values exceeding the predefined threshold of -5.0 kcal/mol. Strikingly, the *He*AR-ATR complex exhibited a remarkably low ΔG of −12.27 kcal/mol, approximately 50% lower than that of the BR-ATR system (−6.12 kcal/mol), suggesting superior retinoid-binding potential in *He*AR (Figure 12). However, molecular docking failed to recapitulate the Schiff base covalent interactions between ATR and conserved lysine residues (*He*AR Lys226, BR Lys216) observed in dark-adapted crystal structures, a discrepancy potentially attributable to simulation constraints inherent in covalent docking protocols. Spatial mapping of residues within 3.5 Å of the retinal chromophore unveiled conserved structural motifs across homologs: AR1 (Asp224/Trp98/Ser153/Pro198/Trp194), AR2 (Thr100/Tyr196/Trp149/Ser152/Trp97), and predicted *He*AR (Trp96/Tyr195/Pro196/Trp148/Ser151) shared a Trp-Tyr-Ser tripartite signature. BR-family members (PDB 1C3W vs. predicted BR) demonstrated evolutionary conservation at Trp86/Tyr185/Trp189/Ser141, residues critical for photocycle-driven proton translocation (Figure 13). Intriguingly, Alphafold-predicted models exhibited expanded interfacial residue participation compared to experimental structures (*He*AR:10 vs. AR1:5; predicted BR:13 vs. crystalline BR:7), potentially reflecting enhanced modeling of conformational flexibility in loop regions. Evolutionary analysis reveals that the AR and BR families, functioning as proton pumps, exhibit significant conservation at Trp/Tyr/Ser residue positions (sequence similarity >86%). This suggests that these amino acids may play crucial roles in maintaining the retinal binding conformation and proton transfer pathways.

### 3.9. Molecular Dynamics Analysis

**Figure 14:**
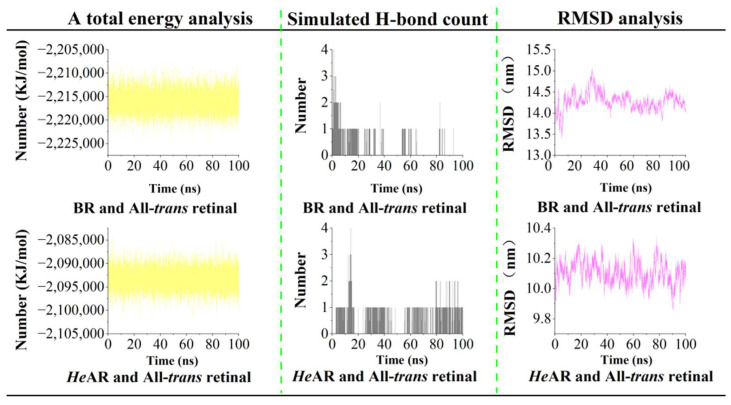
Molecular dynamics simulations of All-*trans* retinal bound to AlphaFold-predicted structures of *He*AR and BR, encompassing: Total energy analysis over the simulation trajectory, demonstrating system stability and energetic profiles; Hydrogen bond analysis, illustrating the dynamic nature and persistence of key interactions between the retinal chromophore and protein residues; Root mean square deviation (RMSD) plots, quantifying structural deviations from initial conformations and elucidating protein flexibility.

**Figure 15:**
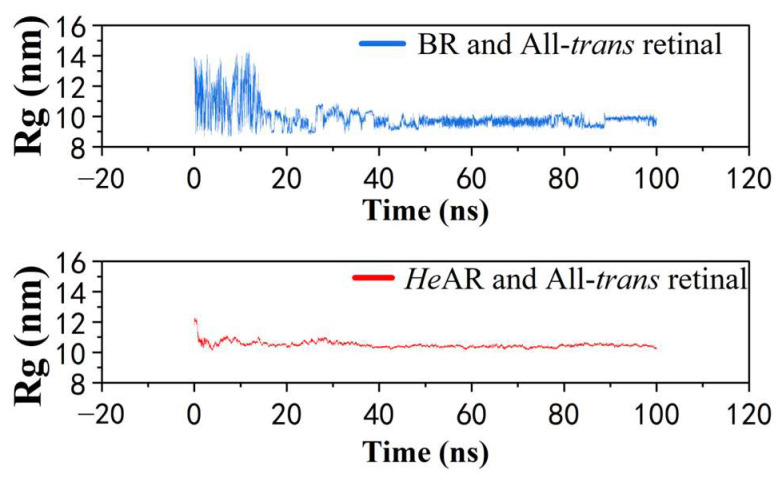
Radius of Gyration (Rg) analysis, providing insights into the overall compactness and conformational changes of the protein-retinal complexes throughout the simulation.

Molecular dynamics simulations of the All-*trans* retinal-bound *He*AR and BR systems revealed distinct structural and energetic signatures between the two homologs (Figure 14). Total energy analysis demonstrated that both systems reached equilibrium states, with *He*AR exhibiting a total energy of −2.1 × 10^6^ kJ/mol and BR showing a marginally lower value of −2.2 × 10^6^ kJ/mol, consistent with their structural divergence. Hydrogen bond dynamics at the retinal-binding pocket displayed notable differences: While both systems maintained an average of 1.0 (±0.5) hydrogen bonds between the chromophore and surrounding residues, *He*AR preserved these interactions with 80% temporal persistence compared to BR’s 40%, suggesting enhanced stabilization of the retinal cofactor in *He*AR. Root mean square deviation (RMSD) trajectories quantified distinct folding pathways: The *He*AR-retinal complex achieved rapid stabilization with an average RMSD of 2.0 ± 0.2 Å throughout the simulation, whereas BR exhibited higher initial flexibility (2.3 ± 0.3 Å) before converging to a stable conformation after 40 ns. Complementary radius of gyration (Rg) analysis corroborated these trends, revealing that *He*AR maintained a compact architecture (10.4 ± 0.1 nm) with minimal fluctuations, while BR underwent transient expansion (9.8 ± 0.2 nm) followed by compaction post-40 ns (Figure 15). These collective metrics—energetic convergence, hydrogen bond persistence, and structural rigidity—position *He*AR as a system with reduced conformational entropy relative to BR, potentially reflecting evolutionary adaptations in retinal-protein coupling mechanisms. The divergent stabilization kinetics implicate critical residue-level interactions governing functional divergence within the microbial rhodopsin family.

## 4. Discussion

This study provides a comprehensive analysis of the *He*AR protein derived from the halophilic archaeon *Halorubrum* sp. Ejinoor, isolated from Inner Mongolian salt lakes. Our findings reveal unique molecular conformations, photodynamic properties, and evolutionary adaptations of *He*AR, while also exploring its potential applications in optogenetics. Here, we integrate our experimental results with existing literature to elucidate the scientific significance of our findings from multiple perspectives.

### 4.1. Molecular Conformation and Structural Stability

*He*AR was successfully expressed in *E. coli* BL21 (DE3) and spontaneously assembled into trimers (SEC analysis: 129 kDa, Figure 2), with circular dichroism (CD) spectral characteristics highly consistent with those of bacterial bacteriorhodopsin (BR) (Figure 3). This similarity suggests that *He*AR and BR share comparable transmembrane helical topologies [52]. However, *He*AR exhibits a 10 nm red-shift in maximum absorption wavelength compared to BR (535 nm vs. 525 nm), and its protonated Schiff base counterion Asp-95 has a significantly higher pKa value (3.5) than BR’s Asp-85 (2.6) (Figure 6). This nearly tenfold increase in proton affinity indicates functional evolution in the electrostatic microenvironment of *He*AR’s retinal binding pocket [38]. AlphaFold predictions reveal that *He*AR and BR form a highly conserved spatial network in the proton transfer core region (e.g., R92/D95/M128 vs. R82/D85/M118, RMSD < 1.3 Å). However, *He*AR’s unique Trp96/Tyr195/Ser151 triad reshapes the rigidity of the chromophore binding cavity through distinctive π-π stacking interactions (Figure 13) [33]. Molecular dynamics simulations further corroborate that the *He*AR-retinal complex exhibits both significantly higher hydrogen bond persistence (80%) compared to BR (40%), and lower conformational fluctuations (RMSD = 2.0 ± 0.2 Å) (Figure 14). These findings suggest that *He*AR optimizes photoenergy conversion efficiency by reducing conformational entropy [53].

### 4.2. Photodynamics and Optogenetic Adaptability

*He*AR demonstrates proton pumping activity (0.1 ng H⁺/mg·s) equivalent to BR, with DCCD enhancement effects revealing a synergistic regulatory mechanism with H⁺-ATPase (Figure 8a). The dark adaptation conversion time of *He*AR (160 min) is significantly longer than that of BR (80 min), and its All-*trans*/13-*cis* retinal isomer ratio (2:1) in the light-adapted state exceeds that of BR (1:1) (Figure 5). These observations indicate an elevated energy barrier for chromophore isomerization [39], consistent with the higher hydrogen bond stability (80% persistence) observed in molecular dynamics simulations (Figure 14). This slow dark-state recovery, coupled with rapid photoactivation (M-state saturation time of 60 μs), creates a “bistable optical switch” characteristic that could potentially address the slow inactivation drawback of optogenetic tools like *Np*HR [54]. Moreover, the *He*AR-ATR complex exhibits significantly higher binding free energy (ΔG = −12.27 kcal/mol) compared to BR (−6.12 kcal/mol). The hydrophobic pocket formed by Trp148/Ser151/Tyr195 may enhance photosensitivity stability through increased van der Waals interactions (Figure 12), a crucial factor for long-term neuronal photocontrol.

### 4.3. Functional Evolution and Cross-Species Comparison

Phylogenetic analysis reveals that *He*AR shares higher transmembrane domain homology (86–95%) with *Halorubrum* genus AR proteins (AR1-AR4) than with bacterial BR (57%). However, its photochemical cycle pathway (L→M→O state sequence) closely resembles that of BR (Figure 7) [55]. The 18 nm difference in absorption spectra between *He*AR and BR (550 nm vs. 568 nm) suggests an evolutionary strategy of spectral differentiation through key site micro-mutations in the *Halorubrum* genus archaea. For instance, the Asp-95 in *He*AR’s proton release cluster (corresponding to BR’s Asp-85) demonstrates enhanced adaptation to proton scarcity in high salt environments through its elevated pKa value (3.5 vs. 2.6). This evolutionary innovation provides new insights for improving the photocontrol precision of optogenetic tools: modulating the electrostatic environment around the Schiff base through site-directed mutagenesis could potentially achieve wavelength-specific optimization similar to the Chronos series tools [56].

### 4.4. Optogenetic Application Prospects

Compared to current mainstream inhibitory tools like AR3 and *Np*HR, *He*AR exhibits three distinct advantages: (1) Its 550–560 nm activation window (Figure 4) has low spectral overlap with excitatory tools such as ChR2 (470 nm) and VChR1 (530 nm), enabling independent multi-channel regulation; (2) The extended dark adaptation half-life of 160 min (Figure 4b) supports sustained hyperpolarization under low-frequency light stimulation, reducing phototoxicity risks; and (3) These properties make *He*AR suitable for constructing “optical switch-memory” systems, potentially regulating membrane potential in chronic neurodegenerative diseases [57]. Compared to BR, *He*AR’s superior stability makes it more suitable for long-term inhibition studies in neural circuit research. Future work could focus on optimizing *He*AR expression through directed evolution [58] or creating fusion constructs with ChR2 to develop bidirectional optical control systems [59], thereby expanding its range of applications.

## 5. Conclusions

This study elucidates the structural and functional properties of *Halorubrum* sp. Ejinoor Archaerhodopsin (*He*AR), a red-shifted proton pump with enhanced photostability and prolonged dark adaptation (160 min). *He*AR’s unique spectral window (550–560 nm), reduced conformational entropy, and elevated proton affinity (Asp-95 pKa = 3.5) position it as a superior optogenetic tool for low-irradiance, long-term neuromodulation. Future engineering efforts should focus on: (1) spectral tuning via site-directed mutagenesis of Trp/Tyr/Ser networks to expand activation wavelengths beyond 600 nm, enabling deeper tissue penetration; (2) kinetic optimization through AlphaFold-guided redesign of the retinal-binding pocket to decouple M/O intermediate lifetimes for millisecond-scale optical control; (3) multiplexed systems integrating *He*AR with excitatory opsins (e.g., ChR2/VChR1) via fusion constructs, enabling bidirectional neural circuit regulation. Additionally, leveraging *He*AR’s ATP synthase compatibility could pioneer optobioenergetic therapies for mitochondrial disorders. These strategies, combined with directed evolution and cryo-EM-guided engineering, will unlock *He*AR’s full potential in precision neuroscience and synthetic biology.

## Data Availability

Data are contained within the article.
